# Key determinates of job satisfaction for acute care nurse practitioners in Taiwan

**DOI:** 10.1186/s12912-022-01156-x

**Published:** 2023-01-05

**Authors:** Sheng-Shiung Huang, Cheng-Yuan Chen, Kevin Kau, Jung-Mei Tsai, Shiow-Luan Tsay

**Affiliations:** 1 College of Nursing and Health Sciences, Da-Yeh University, Changhua, Taiwan; 2 National Taiwan University Hospital Yunlin Branch, Yunlin, Taiwan; 3 Academic Writing Education Center, National Taiwan University, Taipei, Taiwan; 4 Department of Nursing, Mackay Memorial Hospital, Taipei, Taiwan; 5 Department of Nursing, Da-Yeh University, Changhua, Taiwan

**Keywords:** Nurse practitioner, Empowerment, Burnout, Job satisfaction, National survey, Taiwan

## Abstract

**Background:**

Taiwan is a super-aged society, and the shortage of hospital doctors; nurse practitioners (NPs) became vital healthcare providers to fulfill the healthcare demands of the population. The purpose of this study was to explore the key determinates of job satisfaction for NPs in acute care practices using significant practice variables, such as empowerment and burnout.

**Methods:**

Participants of this descriptive survey study were recruited from a national sample of NPs with membership in the Taiwan Association of Nurse Practitioners. The data were collected utilizing an online questionnaire based on demographic and practice variables, the Misener Nurse Practitioner Job Satisfaction Scale (MNPJSS), the Condition for Work Effectiveness Questionnaire II (CWEQ II), and the Copenhagen Burnout Inventory (CBI). A total of 1,211 NPs completed the online survey. A multiple regression model with the stepwise selection was used to explore job satisfaction.

**Results:**

The mean overall satisfaction score indicated that the level of satisfaction was between slightly dissatisfied and slightly satisfied. Regression results indicated that formal power, work-related burnout, access to information, and needed resources were critical components of job satisfaction, and accounted for 63% of the variance. Moreover, NPs who were married, had a higher annual salary, worked only during the day shift, and had lower patients-related burnout showed better job satisfaction.

**Conclusions:**

This study provides evidence for healthcare organizations to formulate policies to strengthen NP job satisfaction. Empowerment and burnout are vital factors in NPs’ job satisfaction. Healthcare organizations have an obligation to implement policies to empower NPs in practice and provide interventions to mitigate burnout. Implementing these changes will improve job satisfaction and with it the quality of patient care.

## Introduction

In March 2018, Taiwan officially became an aged society—over 7% of the total population being over 65 years old. This trend is not slowing down. By 2025, the population aged above 65 is estimated to exceed 20% and become a “super-aged” society [1]. Demands for health care will continue to increase rapidly despite Taiwan already experiencing a shortage of trained physicians and residents. The Ministry of Labor recently made matters worse in 2019, when it reduced weekly working hours for physicians and medical residents from 88 to 80 h [2]. Thus, the shortage of healthcare workers could turn out to be an even more severe issue in the near future.

### Nurse practitioner roles in Taiwan

The Taiwan government, after several rounds of consultations, officially decided to establish the nurse practitioner (NP) program as a response to the growing healthcare needs of older population [3, 4]. This program allows an experienced registered nurse that receives standardized professional theory and clinical training, and also passes the NP licensure examination, to provide clinical health care to acute and chronic patients. Many hospitals have employed NPs to team up with physicians to deliver health care to patients, thus, helping to alleviate the shortage of physicians [5]. The standard of care has not fallen with the addition of more NPs. A recent retrospective cohort study found that the practice outcomes and the quality of care provided by NPs were similar to physicians [6]. NPs have been part of an effective solution in the unburdening of Taiwan’s healthcare system. Nevertheless, the job descriptions of NPs were determined by the hospital demand in Taiwan [4]. Because the Physician Act of Taiwan prohibits other healthcare professionals from diagnosing, prescribing medications, and treating patients, therefore, NPs have to team up and collaborate with physicians in clinical practice. With such restricted practice, NPs are routinely taking care of patients using authorized guidelines. Therefore, the factors influencing NP practice are incredibly worthy to explore, especially job satisfaction.

### NPs’ job satisfaction

Job satisfaction is a dynamic multidimensional concept that is comprised of NPs’ expectations, values, practice environment, and personal characteristics [7]. Though the scope of practice regulations for NPs in Taiwan had been formulated in 2016, their clinical practice experience has varied by hospitals. NPs might perceive less support from the organization and show lower job satisfaction [8]. According to recent studies from a national survey in Taiwan, NPs’ job satisfaction levels in hospitals ranged from unsatisfied to slightly satisfy [9, 10]. The results of Ho et al. (2021) further noted that insufficient organizational support could lead to lower job satisfaction among NPs in hospitals. On the contrary, Poghosyan et al. (2020) found that increased empowerment support from organizations was positively associated with NP job satisfaction. Kanter (1993) indicated that empowerment was the ability provided by organizations for employees to mobilize resources such as information, the opportunity to develop, and support to complete their job in the organization [11]. Importantly, Dillon and colleagues (2016) research emphasized that organizational support, communication, and leadership were essential for ACNP practice [12]. In other words, if the organization provided orientation or fellowship programs and sufficient support for NPs, then the NPs experienced better job satisfaction, a higher probability of retaining their position, and delivering an enhanced quality of care [8]. From this point of view, adequate empowerment could be a critical factor for promoting NPs’ job satisfaction in practice. Another possible consideration would be adoption of a ‘credentialing committee’ for NPs upon hire. This would delineate their specific privileges as well as show administrative support for their role.

Studies support that increased job satisfaction could reduce turnover and produce a positive influence on retention. For example, Horner (2020) noted that NPs showed lower turnover with better patient outcomes when they have a higher level of job satisfaction [13, 14]. Similarly, Auffermann et al. (2020) indicated that practice conditions and the intent to leave their current position were related to NPs’ job satisfaction [15]. Additionally, Lu et al. (2019) also concluded that the improvement of job satisfaction is important to ensure both the quality of care and an adequate nursing workforce [16]. The literature shows that it is vital to improving job satisfaction for maintaining NP retention. This is crucial for decreasing the impact of the healthcare labor-force shortage and sustaining the quality of care for Taiwan’s soaring aged population.

### Empowerment and job satisfaction

Numerous studies have reported that better empowerment could increase NPs’ job satisfaction [17, 18]. However, these studies were mostly conducted with a small sample size and data were collected from only a few hospitals. To the best of our knowledge, only one recent study conducted in Taiwan used a national survey to indicate that higher empowerment was correlated with job satisfaction [14]. This study, however, focused on empowerment as a whole and did not investigate individual dimensions of empowerment. It might be more effective to develop exclusive interventions according to specific needs of empowerment in promoting NPs’ job satisfaction in practice.

### Burnout and job satisfaction

According to several recent studies, burnout is another likely and important factor in NPs’ job satisfaction [14, 19, 20]. Burnout is defined as a state of chronic physical, mental or emotional exhaustion, which leads to long-term stress at work [21]. Burnout manifests itself through symptoms of exhaustion, cynicism, and diminished professional efficacy. The result of burnout is mainly associated with ill-health and decreased work ability that then negatively affects the quality of patient care [21].

Healthcare in the acute setting is an intense environment with higher stress, where NPs provide health care to patients and such stress could contribute to burnout [22]. Burnout is a common phenomenon among healthcare professionals. Recent studies estimate that 25.3%-33.3% of healthcare workers suffer from burnout [20, 23, 24]. Likewise, clinician burnout has been a critical issue for the healthcare system as it has elicited several negative outcomes. For instance, it has been proven that burnout not only had negative effects on job satisfaction [14, 25] but also related to lower quality of care [23] and a higher likelihood of NPs intention to leave [24]. Additionally, several studies also found a negative influence of burnout on job satisfaction. Friganović et al. (2019) recently conducted a systematic review of intensive care nurses and concluded that burnout was negatively associated with increased job satisfaction [26]. The results of Ran et al. (2020) echoes this with their study finding that burnout had negative effects on job satisfaction among primary healthcare staff [14]. A similar study by Alharbi et al. (2016) found burnout in critical care nurses to be a predictor of poor job satisfaction [27]. Hoff et al. (2019) indicated that NPs in the current healthcare system frequently faced more demanding jobs and tasks in practice, which could contribute to burnout [19]. To date, detailed information on the burnout and the association between burnout and job satisfaction among NPs has not been fully examined by a national sample in Taiwan. Therefore, exploring different sources of burnout among NPs, and the resulting need to mitigate these influences of burnout on job satisfaction, should be thoroughly investigated.

### Demographic and practice characteristics associated with job satisfaction

An NP’s demographic characteristics may also influence job satisfaction. Atefi et al. (2015) documented that young, female, married individuals with more experience of practice reported a higher level of job satisfaction [28]. In addition, several studies have reported better job satisfaction among nurses and NPs with a higher educational level that worked the day shift and in a friendlier environment [29–31].

Practice characteristics were also found to have an effect on job satisfaction. For instance, recent studies confirmed that a higher patient load and longer working hours could decrease job satisfaction [9, 32]. One study showed that a higher annual salary also increased job satisfaction [10]. So, it is also necessary to include the influence of specific demographic and practice characteristics when examining the impact of empowerment and burnout on NPs job satisfaction.

In summary, there is evidence to indicate that empowerment and burnout are associated with job satisfaction. Yet, majority of studies in the literature used small sample size, so we still need to verify the relationships among these variables with a representative sample, and then explore how these relationships of empowerment and burnout might impact NPs’ job satisfaction. Thus, the purpose of this study is to assess the associations between empowerment, burnout, and job satisfaction among all NPs, and to explore the potential impact of demographic characteristics and the distinct dimensions of empowerment and burnout associated with NPs’ job satisfaction.

## Methods

### Design and participants

This survey study utilized the Taiwan Association of Nurse Practitioners database in order to access all 7,046 active members and invite those qualified to participate in the study from July 1 through August 30, 2021. Because the vast majority of NPs in Taiwan work in hospital settings. The study’s inclusion criteria limited the participants to NPs who had a national NP certification and worked in an acute care setting for at least one year. All qualified 6,154 eligible NPs were approached via an e-mail invitation that included information of this study such as the purpose, procedures, and a uniform resource locator (URL) of the online survey. We contacted eligible NPs twice during the recruiting phase of the research. The eligible NPs who agreed to participate filled out a consent form and proceeded to finish the online self-rated questionnaires. Instruments included demographic and practice information, job satisfaction, empowerment, and burnout measures. All procedures of this study were approved by the Institutional Review Board of hospitals in Taiwan (Approval NO: 202012178RINA).

Of the 6,154 eligible NPs, there were a total of 1,211 NPs that completed the online survey, producing a 19.68% response rate for the study. The sample size was estimated with the G*Power Software Version 3.1.9.7 and the criteria for multiple logistic regression analysis were as follows: power = 0.95, α = 0.05, effect size (f^2^) = 0.15, and the number of predictors = 10. Additionally, we estimated a 30% attrition rate in the study with a minimum sample size of 224 for effective analysis. Thus, our 1,211 NP participants satisfied the basic sample size requirement.

### Measurement

#### Demographic characteristics

NPs’ demographic and practice information were obtained from the self-rated questionnaire. This information included gender, marital status, education level, care model in practice, work in shift, hospital level, age, the experience of NP (in years), NP advancement level, working hours, daily patient load, and annual salary. The nurse practitioner clinical ladder advancement was based on the standard of the Taiwan Association of Nurse Practitioners. Each hospital adopted the program and compensated for each ladder with an additional subsidy.

#### Job satisfaction

NPs’ job satisfaction was measured with the Misener Nurse Practitioner Job Satisfaction Scale (MNPJSS). This scale was developed for assessing NPs’ job satisfaction in practice. It contained 44 items and included six factors: intrapractice partnership/collegiality; challenge/autonomy; professional, social and community interaction; professional growth; time; and benefits. Each item was rated on a six-point scale ranging from very dissatisfied [1] to very satisfied [6, 11]. The total score was calculated by summing all items with a higher score indicating a higher level of satisfaction. This scale has been supported to have acceptable reliability and validity in previous studies [11, 14]. The Cronbach's alpha value of this study was 0.93.

#### Empowerment

Empowerment was measured with the Condition for Work Effectiveness Questionnaire II (CWEQ-II). It was developed to measure the organizational support of NPs’ practice environment [33]. It consisted of 19 items and each item was rated on a five-point scale from strongly disagree [1] to strongly agree [5]. This questionnaire included six subscales: opportunity, information, resources, support, formal power, and informal power. ‘Opportunity’ refers to the opportunity for growth within the organization. ‘Information’ implies having the professional knowledge that is essential to the effectiveness of work. ‘Resources’ indicate an individual’s ability to access the resources associated with work. ‘Support’ concerns the perception of feedback from work. ‘Formal power’ refers to the flexibility associated with decision-making or visibility at work. ‘Informal power’ relates to the development of communication with managers or colleagues at work. The scores of each subscale were obtained by summing the subscale items and averaging the items [33]. The scores ranged from 1 to 5 with a higher score corresponding to stronger empowerment at their job. Previous researchers have been satisfied with both reliability and validity using this methodology [14, 33]. Cronbach's alpha for the total scale was 0.95 in this study.

#### Copenhagen burnout inventory

Burnout status was assessed using the Copenhagen Burnout Inventory [34]. The inventory consisted of three domains including personal burnout, work-related burnout, and clients-related burnout. These domains evaluate a participant’s prolonged physical and psychological exhaustion as related to the individual, the work environment, and the work associated with clients, respectively. The inventory was composed of 19 items rated on a scale from never (0) to always (100) with an interval of 25 points. The total score on each subscale was calculated by averaging the scores on the items. Higher scores indicate greater levels of burnout experienced by NPs. The scores under 50 represented low to no burnout. Scores ranging from 50 to 74 were viewed as moderate burnout and scores above 75 were considered high burnout. Previous research has demonstrated the acceptable reliability and validity of this measure [34, 35]. The Cronbach's alpha of total inventory was 0.95 in the current study.

### Statistical analysis

Descriptive statistics were performed to examine the distribution of NPs such as number (n), percentage (%), mean, and standard deviation (SD). An independent t test was used to examine the significant differences between demographic characteristics and job satisfaction. The correlation between continuous variables, empowerment, burnout, and job satisfaction was examined by the Pearson product-moment correlation coefficient (*γ*). Multiple regression analysis was performed to examine the effects of demographic characteristics, empowerment, and burnout on job satisfaction. The multiple regression included the identified significant variables associated with job satisfaction and the stepwise selection method detected variables associated with job satisfaction. The variance inflation factor (VIF) was utilized to examine the multicollinearity in multiple regression. If the VIF value of variables exceeded 3, then it indicated that there was multicollinearity in the model; therefore, the variable would be excluded from the model [36]. The statistical significance level was 0.05 with a two-tailed test. Statistical Package for Social Sciences (SPSS) software version 22 was used for all analyses.

Of the 1,211 NPs, there were 155 participants with missing data in the MNPJSS; nevertheless, the missing rate (12.80%) was within an acceptable range [37]. We utilized the Markov chain Monte Carlo (MCMC) multiple imputation procedure to impute the missing values in the MNPJSS. The MCMC algorithm was suitable for data with complex patterns of missing data and it has good convergence properties to ensure the imputed data can further be analyzed appropriately [38]. Also, results of sensitivity analysis indicated that there were not statistically significances (*p* > 0.05) between original data and imputed data in job satisfaction.

## Results

### Descriptive analysis results

The average age of NPs was 42.39 years (SD = 6.16). The majority are female (94.10%), married (65.81%), hold a college or postgraduate degree (100%), worked day shift (70.90%), and in a community hospital (63.00%). Fifty-eight percent of NPs were teamed up with attending physicians to provide patient care, and other NPs worked with MD and resident teams (42.12%). The average experience of NP was 8.28 years (SD = 4.45). The majority of NP advancement level was level 2 (*n* = 308, 25.4%) or level 3 (*n* = 431, 35.6%). NPs worked 9.16 h a day on average (SD = 1.25), and cared for an average of 13.35 patients daily in acute care settings (SD = 8.16) (Table 1).

**Table 1:** Demographic characteristics and correlations with job satisfaction (*N* = 1,211)

Variable	*n*	%	Mean	*SD*	*t/r*	*p* value
Age			42.39	6.16	0.11	< 0.001
Gender						
Female	1140	94.10	172.62	32.12	-0.32	0.75
Male	71	5.90	171.10	38.98		
Marital status						
Married	797	65.81	175.02	31.91	3.71	< 0.001
Unmarried	414	34.19	167.73	33.25		
Education degree						
College/University	949	78.36	172.03	31.60	0.93	0.35
Graduate degree^a^	262	21.64	174.31	35.73		
Care model						
MD + NP	713	58.88	169.11	32.65	4.40	< 0.001
MD + Resident + NP	498	41.12	177.43	31.78		
Hospital level						
Non-medical center^b^	763	63.00	170.14	31.30	3.34	0.001
Medical center	448	37.00	176.60	33.04		
Day shift						
Yes	859	70.90	173.93	31.12	2.37	0.02
No	352	29.10	169.04	33.36		
Experience of NP (years)			8.28	4.45	0.08	< 0.01
NP advancement level			2.52	1.22	0.17	< 0.001
Working hour (per day shift)			9.16	1.25	-0.12	< 0.01
Patient load (per day shift)			13.35	8.16	-0.08	< 0.01
Annual Salary (10,000 NTD)			79.66	17.14	0.15	< 0.001
Job satisfaction			172.52	32.54		
Empowerment						
Opportunity			3.32	0.66		
Information			3.13	0.74		
Resources			2.91	0.79		
Support			3.11	0.76		
Formal power			2.94	0.75		
Informal power			3.33	0.69		
Burnout						
Personal burnout			50.92	19.97		
Work-related burnout			44.62	17.96		
Patients-related burnout			34.37	16.60		

*SD* Standard Deviation, *NTD* New Taiwan dollar

^a^Including master and doctorate degree

^b^Including community and regional hospitals

The mean scores of total job satisfaction were 172.52 (SD = 32.54) and the mean overall satisfaction score was 3.92, indicating that the level of satisfaction was slightly dissatisfied to slightly satisfied. For empowerment, the mean score for opportunity was 3.32 (SD = 0.66); information, 3.31 (SD = 0.74); resources, 2.91 (SD = 0.79); support, 3.11 (SD = 0.76); formal power, 2.94 (SD = 0.75); and informal power, 3.33 (SD = 0.69) which indicate that NPs received a intermediate level of empowerment from the hospital. The mean score of burnout in each subscale was less than 50, which indicate that NPs had a lower level of burnout. The mean scores for personal burnout, work-related burnout, and patients-related burnout were 50.92 (SD = 19.97), 44.62 (SD = 17.96), and 34.37 (SD = 16.60), respectively. There were 27.3% of NPs that had moderate burnout (mean = 63.21, SD = 11.03).

### Correlations between demographic characteristics, job satisfaction, empowerment, and burnout

Table 1 demonstrates the correlations between demographic characteristics and job satisfaction. The marital status, care model, hospital level, day shift, and age were significantly associated with job satisfaction (*p* < 0.05). The experience of NPs and their annual salary were positively correlated with job satisfaction (*r* = 0.08–0.17, *p* < 0.01). Though, patient load (*r* = -0.08, *p* < 0.01) and work hours (*r* = -0.12, *p* < 0.01) had a significantly negative association with job satisfaction.

All the subscales of empowerment were positively correlated with job satisfaction (*r* = 0.58–0.70, *p* < 0.01). In contrast, personal burnout (*r* = -0.46, *p* < 0.01), work-related burnout (*r* = -0.50, *p* < 0.01), and patient-related burnout (*r* = -0.37, *p* < 0.01) showed negative correlations with job satisfaction (Table 2).

**Table 2:** The correlations between empowerment, burnout, and job satisfaction of NPs (*N* = 1,211)

**Variables**	1	2	3	4	5	6	7	8	9	10
1. Job satisfaction	-									
2. Opportunity	0.58^**^	-								
3. Information	0.69^**^	0.70^**^	-							
4. Resources	0.67^**^	0.51^**^	0.61^**^	-						
5. Support	0.69^**^	0.68^**^	0.85^**^	0.67^**^	-					
6. Formal power	0.70^**^	0.63^**^	0.71^**^	0.65^**^	0.76^**^	-				
7. Informal power	0.58^**^	0.66^**^	0.67^**^	0.57^**^	0.67^**^	0.66^**^	-			
8. Personal burnout	-0.46^**^	-0.23^**^	-0.25^**^	-0.42^**^	-0.29^**^	-0.29^**^	-0.30^**^	-		
9. Work-related	-0.50^**^	-0.30^**^	-0.31^**^	-0.44^**^	-0.33^**^	-0.33^**^	-0.36^**^	0.89^**^	-	
10. Patients-related	-0.37^**^	-0.27^**^	-0.22^**^	-0.23^**^	-0.22^**^	-0.21^**^	-0.34^**^	0.47^**^	0.56^**^	-

^**^:*p* < 0.01

### Potential factors associated with job satisfaction

Formal power (β = 0.30, *p* < 0.001), work-related burnout (β = -0.17, *p* < 0.001), information (β = 0.24, *p* < 0.001), and resource (β = 0.21, *p* < 0.001) critically affected job satisfaction among NPs, which accounted for 63% of the variance (F = 65.70, *p* < 0.001). Moreover, NPs who were married (β = 0.04, *p* < 0.05), who had higher annual salary (β = 0.06, *p* < 0.01), and who worked on day shift (β = 0.04, *p* < 0.001) reported higher job satisfaction. However, a higher patients-related burnout (β = -0.10, *p* < 0.001) decreased job satisfaction (Table 3).

**Table 3:** Results of stepwise multiple regression in job satisfaction of NPs (*N* = 1,211)

Variables	Regression coefficient			Model summary		
	**B**	**SE**	**β**	**ΔR**^**2**^	**Adj-R**^**2**^	**F**
Intercept	82.85	4.66	-	-	-	-
Empowerment-Formal power	13.20	1.17	0.30^***^	0.48	0.48	1109.19^***^
Work-related burnout	-0.31	0.04	-0.17^***^	0.08	0.56	224.72^***^
Empowerment-Information	10.33	1.12	0.24^***^	0.05	0.61	142.06^***^
Empowerment-Resource	8.72	1.02	0.21^***^	0.02	0.63	65.70^***^
Patients-related burnout	-0.19	0.04	-0.10^***^	0.006	0.63	19.62^***^
Marital status (ref = unmarried)	2.92	1.20	0.04^*^	0.003	0.64	10.63^**^
Annual Salary (10,000 NTD)	0.12	0.03	0.06^**^	0.002	0.64	6.96^**^
Day shift (ref = no)	3.03	1.26	0.04^***^	0.002	0.64	5.76^*^

*B* Unstandardized regression coefficient, *SE* Standard Error, *β* Standardized regression coefficient

^*^p < 0.05; ^**^p < 0.01; ^***^p < 0.001

The results of stepwise multiple regression revealed that 64% of the variance in NPs’ job satisfaction was explained by empowerment, burnout, marital status, annual salary, and day shift (F = 5.76, *p* < 0.05). The VIF for support was above 4; therefore, it was excluded because of the multicollinearity issue in this model and in which all other VIF were quite small (Table 3).

## Discussion

The purpose of this study was to identify the effects of different dimensions of empowerment and distinct types of burnout in regard to job satisfaction among NPs in Taiwan. The results revealed that NPs’ job satisfaction ranged from slightly dissatisfied to slightly satisfied. Moreover, different dimensions of empowerment such as formal power, information, and resources were the main positive influential factors associated with job satisfaction, which could explain 55% of the variance in job satisfaction. In contrast to empowerment, work-related burnout and patient-related burnout had negative effects on job satisfaction, which could explain 8.6% of the variance in job satisfaction. Furthermore, some specific demographic characteristics were associated with job satisfaction. For example, NPs who were married, have a higher annual salary, and worked day shift reported better job satisfaction.

### Empowerment effects on job satisfaction

Our results indicate that increased empowerment improved NPs’ job satisfaction, which is consistent with findings from previous studies [8, 10, 18]. The results also revealed that different dimensions of empowerment positively influenced job satisfaction, and that formal power was the main factor influencing NPs’ job satisfaction. It is logical that greater access to formal power could enhance job performance, which then promotes higher job satisfaction [39]. However, in Taiwan, NPs’ roles and functions varied according to their healthcare organizations’ demands. Because of this variance, NPs’ practice may not be equivalent to their functional role, which could contribute to ambiguity and role conflict. For example, NPs are not able to practice to the full extent of their licensure and or education. NPs might feel frustrated in their practice when inadequately empowered and exhibit lower job satisfaction when their ability was limited. It has also been proven that a higher level of ambiguity and role conflict causes a lower level of job satisfaction [40, 41]. Similarly, Orgambídez and Almeida (2020) also noted that empowerment could decrease the negative effects of role ambiguity (β = -0.58, *p* < 0.01) and role conflict (β = -0.48, *p* < 0.01) and increase job satisfaction (β = 0.74, *p* < 0.001) [18]. Also, Sureda et al. (2018) noted that the prevention of psychosocial risk, such as solidifying role definition, can be an efficient way to increase job satisfaction [41]. Therefore, healthcare organizations should develop a reasonably uniform guideline to identify the responsibilities and duties within the NPs’ scope of practice in order to support role clarity and avoid ambiguity and role conflict. Déry et al. (2018) also noted that enacting a clear scope of nursing practice could decrease role ambiguity and improve job satisfaction [40]. Furthermore, their practice is complex, diverse, and urgent, which might increase the risk of lawsuits [42]. Because of this, healthcare organizations might tend to set more restrictions on individual NPs’ practice to avoid medical malpractice claims, especially when NPs perform invasive treatment or procedures. Unlike physicians, NPs and managers were more concerned with having clear regulations to protect their practice [4]. A clear legislated scope of practice could positively impact NPs’ practice [43].

Based on our study results, we suggest that NPs need to clarify their specific privileges as well as administrative support required for their clinical practice during their employment. Acute care healthcare organizations further empower NPs in clinical practice. NPs would be more recognized for their role and function, leading to improved job satisfaction. Likewise, the government and professional communities ought to work together to establish a practical scope of practice. Particularly, our NPs’ scope of practice is based on the “Nurse Practitioner Practice Act” which restricts practice due to the Physicians Act. Therefore, we need to work collaboratively with physicians, officers, lawmakers, and legislators to expand our scope of practice more independently. In doing so, the collaborative relationships between NPs and physicians could be enhanced, while maximizing the effectiveness of NPs’ role and function, promoting job satisfaction, and providing a high quality of care to the aged population.

We also found that empowerment of information could increase job satisfaction. This is likely due to empowerment of information, including professional knowledge and expertise, being essential to effectiveness in the NP workplace [44]. The results of Faris et al. (2010) suggested that it is necessary to provide strategies for improving professional growth to enhance job satisfaction [45]. A study by Arthur et al., (2020) also supports that NPs value continuing education from colleges and organizations, and that professional advancement ladders were critical for advanced practice nurses to be recognized for their contribution to the health care organizations [46]. Expanding these opportunities could increase their professional identity and their level of job satisfaction. One recent review concluded that continuing educational intervention for nursing needed to be further developed with the growing demands for age care [47]. It is necessary for healthcare organizations to offer continuing education and training programs about the knowledge and skills related to the care of aged population, specifically polypharmacy issues in the geriatric population. With adequate opportunity for professional growth, NPs could provide diversified demands of healthcare organizations, thereby resulting in a higher level of job satisfaction.

Additionally, our results found that more resources of empowerment could increase job satisfaction. The nature of NPs’ work highlights the importance of resources. NPs’ work in acute care is complex as they need extensive resources required for practice, such as materials, time, supplies, and workforce. One recent study has reported that increased job resources could moderate the relationship between job demands and job strain [48]. In other words, NPs with greater empowerment in resources could more smoothly and efficiently provide healthcare for patients. Subsequently, NPs could have better performance in practice and better job satisfaction. Accordingly, our results suggest that managers should empower NPs to have more access to resources related to healthcare. This effort could be a benefit for the reduction in job strain and improvement in job satisfaction.

### Burnout on NPs’ job satisfaction

We also found that burnout had negative effects on job satisfaction, which was in line with previous studies [14, 25]. Though, the scores of each subscale in burnout showed a low level of burnout among NPs, burnout still significantly decreased the level of job satisfaction. A moderate level of burnout affected 27.3% of the NPs in our study; a rate similar with previous studies [23, 24, 42]. We found that work-related and patient-related burnout significantly decreased job satisfaction. For the work-related burnout, this might directly relate to patient load. There were no guidelines for patient load nationally and hospital policies varied. Some hospitals required NPs to rotate shifts, which further increased patient load and decreased job satisfaction. This dynamic is supported by our data which showed that NPs who worked in varying shifts reported a significantly higher score in both work-related (t = 2.92, *p* < 0.01) and patient-related burnout (t = 2.79, *p* < 0.01) than those who did not work in shifts. Similar findings were also confirmed by the multiple regression results of our study, which found that shift work could decrease job satisfaction. The longer work hours also had a negative influence on job satisfaction, quality of care, and patient safety [29, 49]. The role of expansion accompanied by more workload and job demands in practice also led to burnout [19].

NPs suffer serious stress from their jobs. This stress can lead to frustration at work and having insufficient energy when they are with their family. Consequently, NPs reported a lower level of job satisfaction. NPs are working regular shifts, scheduled according to the demands of hospitals in Taiwan. Generally, most NPs work day shift. Yet, there were some NPs that needed to work the night shift as required by hospitals. If hospitals preliminarily assign shift work to the NPs who have a lower willingness to work on particular shifts, NPs showed lower job satisfaction. Healthcare organizations could preliminarily assign shift work to the NPs who voluntarily choose to work on shift so as to increase NPs’ job satisfaction [50]. Except for the adjustment of workload, healthcare organizations should supplement an adequate clinical workforce and formulate clear guidelines to ensure that the functional role of the NP could be fully conducted. Then, NPs might decrease the odds of burning out and have better job satisfaction.

Patients-related burnout was also negatively associated with job satisfaction in our study. Compared to work-related burnout, patients-related burnout was focused on the stress from patients. For example, patients-related burnout includes difficultly communicating with patients, less feedback from patients, and being tired of caring for patients [34]. Burnout might not only depend on workload, but rather on the contents of duty, stress of practice, and the process of care [51]. It is important to develop personal coping strategies to mitigate stress and burnout [22, 52]. Healthcare organizations could provide personal training of effective coping strategies to reduce the negative effects of caring for patients and interaction with patients. For example, undesirable communication, such as verbal aggressiveness, was seen to negatively impact burnout [53]. On the contrary, utilizing healthier communication could help to alleviate burnout [54]. Hence, managers should enhance training in the skills of healthy communication and specifically avoid an aggressive verbal tone and language in order to reduce patients-related burnout and promote NPs’ job satisfaction. Also, the healthcare organizations should provide communication channels for NPs to discuss the stress they are experiencing in practice. This customized psychological consultation would promote an NP’s well-being and increase job satisfaction.

We also found several characteristics associated with job satisfaction. For example, NPs who were married or who did not work shifts showed higher job satisfaction, which was in line with previous studies [29, 55]. The married NPs might face conflicts between family relationships and work, but a higher level of work-family balance could alleviate stress [52]. Hence, NPs perceived less conflicts or stress from work when they experienced a better family relationship. In addition, annual salary also had a positive effect on job satisfaction. A reasonable salary could help NPs not only meet their basic daily need but also fulfill their demand for a better life. Moreover, a higher salary could also be viewed as positive feedback for their contribution to the organization, which provides higher-level needs satisfaction [56].

### Limitations

Although this study showed the critical effects of both empowerment and burnout on job satisfaction with a national survey, this study has two limitations. First, this study was performed with a cross-sectional design, which did not allow us to conduct causal inference; further research with longitudinal design is warranted. Second, this study collected data by an online survey, which might lead to a self-selection bias. However, the participants were recruited from the Taiwan Association of Nurse Practitioners, which included over 90% of the NPs in Taiwan. Furthermore, there was no difference between non-participating and participating samples in the key characteristics. For example, both non-participating and participating NPs had a national NP certification and worked in an acute care setting for at least one year. Hence, the effects of self-selection bias would be conservative. Under these limitations, our findings still revealed that influential factors such as empowerment and burnout are associated with job satisfaction. This would help to establish customizing policies or strategies about enhancing empowerment and decreasing burnout for promoting job satisfaction in practice.

## Conclusions

Empowerment and burnout are vital factors impacting NPs’ job satisfaction. Healthcare organizations have an obligation to implement policies to empower NPs in practice and provide interventions to deal with burnout, so that job satisfaction and quality of patient care can be improved. This study provided evidence that recommends healthcare organizations need to formulate policies to strengthen job satisfaction of NPs. To enhance working empowerment, organizations need to provide formal power, share information and make resources available for NP practice. For burnout, workplace interventions including reduction of work demands, enhancement of decision making, and improving the social support climate might be promising for preventing burnout and emotional exhaustion. Additionally, healthcare organizations should continue to improve practice environments of NPs, such as adjusting salary and allowing more flexibility in work shifts. Caring about NPs’ preferences will lead to enhanced job satisfaction and patient care.

## Data Availability

The data that support the findings of this study are available from the corresponding author upon reasonable request.
